# BPPRC database: a web-based tool to access and analyse bacterial pesticidal proteins

**DOI:** 10.1093/database/baac022

**Published:** 2022-04-04

**Authors:** Suresh Panneerselvam, Ruchir Mishra, Colin Berry, Neil Crickmore, Bryony C Bonning

**Affiliations:** Department of Entomology and Nematology, University of Florida, Gainesville, FL 32611, USA; Department of Entomology and Nematology, University of Florida, Gainesville, FL 32611, USA; School of Biosciences, Cardiff University, Cardiff CF10 3AX, UK; School of Life Sciences, University of Sussex, Brighton BN1 9QG, UK; Department of Entomology and Nematology, University of Florida, Gainesville, FL 32611, USA

## Abstract

Pesticidal proteins derived from the bacterium *Bacillus thuringiensis*, have provided the bases for a diverse array of pest management tools ranging from natural products used in organic agriculture, to modern biotechnological approaches. With advances in genome sequencing technologies and protein structure determination, an increasing number of pesticidal proteins from myriad bacterial species have been identified. The Bacterial Pesticidal Protein Resource Center (BPPRC) has been established to provide informational and analytical resources on the wide range of pesticidal proteins derived from bacteria that have potential utility for arthropod management. In association with a revised nomenclature for these proteins, BPPRC contains a database that allows users to browse and download sequences. Users can search the database for the best matches to sequences of interest and can incorporate their own sequences into basic informatic analyses. These analyses include the ability to draw and export guide trees from either whole protein sequences or, in the case of the three-domain Cry proteins, from individual domains. The associated website also provides a portal for users to submit protein sequences for naming. The BPPRC provides a single authoritative source of information to which all stakeholders can be referred including academics, government regulatory bodies and research and development personnel in the industrial sector. The database provides information on more than 1060 pesticidal proteins derived from 13 species of bacteria, including insecticidal activities for a subset of these proteins.

**Database URL**: www.bpprc.org and www.bpprc-db.org/

## Introduction

The diverse pesticidal proteins produced by bacteria such as *Bacillus thuringiensis* (Bt), *Lysinibacillus sphaericus, Photorhabdus spp*. and *Xenorhabdus spp.,* provide a valuable resource for suppression of insect pest populations (1–3). For example, sprays composed of sporulated Bt have not only been applied for pest control in organic agriculture but also have been used to suppress disease-carrying vectors such as mosquitoes and blackflies (4). Furthermore, crop plants have been engineered by the introduction of bacteria-derived genes expressing pesticidal proteins, rendering them resistant to several pests of major agricultural importance (5–7). The success of this approach for the suppression of insect pests has led to sustained interest in the deployment of bacteria-derived pesticidal proteins for pest management (8). In addition to their utility in crop protection and management of insect vectored diseases, a subset of the proteins, known as parasporins, have activity against certain human cancer cell lines (9).

The bacterial pesticidal proteins used for pest control are environmentally benign and are safe to humans and non-target organisms due to their narrow host range (8, 10). The economic success of bacterial pesticidal proteins has led to the sequencing of a large number of bacterial genomes in the search for proteins with new or enhanced activities. With the advancement of improved protein structure determination and prediction technologies, it has become evident that bacteria-derived pesticidal proteins are structurally diverse (11) and that the 1998 nomenclature system, which did not reflect structural relationships (12), limited appreciation for this diversity. As a result, we published a revised nomenclature to reflect diverse protein folds while maintaining relationships with the previous system (11).

We present the Bacterial Pesticidal Protein Resource Center (BPPRC) as an authoritative resource to allow users to access and analyse these proteins and to characterize their own sequences. The resource is available both online and as portable code and has been designed to facilitate R&D activities within academia and industry as well as facilitate some of the checks required for new product registration. This database is of particular importance for comparing newly discovered bacteria-derived insecticidal proteins with pesticidal proteins that have been commercialized and will facilitate data acquisition for both researchers and regulatory bodies.

## BPPRC overview

**Figure 1.**
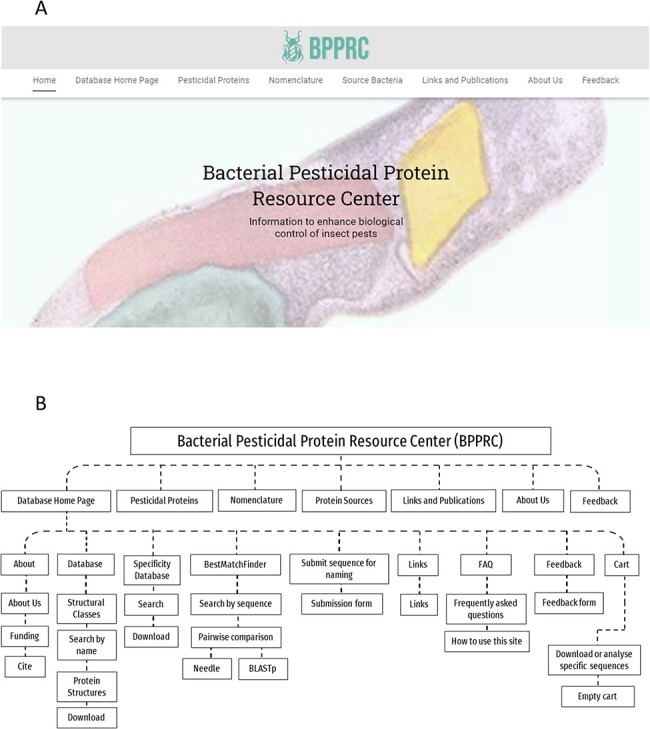
BPPRC website. A. BPPRC home page. B. Schematic of structure and utility of the website.

The BPPRC has been created to develop information systems for the broad range of proteins potentially available for pest control and provides a range of functionalities to inform researchers, regulators and other stakeholders. BPPRC replaces the existing Bt toxin nomenclature site (12) that provided an accepted framework for the naming of proteins used by industry and academia within the field of arthropod management. The BPPRC is an interactive platform that provides authoritative data on bacteria-derived pesticidal proteins. The home page of the site provides links to the main database, descriptions of the various structural classes of proteins, information on the nomenclature system and on the bacterial species from which the genes were identified (Figure 1). Figure 1 also provides a schematic overview of the website structure and utility.

## Source data

The data used were initially extracted from the previous Bt toxin nomenclature page http://www.lifesci.sussex.ac.uk/home/Neil_Crickmore/Bt/. These sequences have been supplemented by a range of other invertebrate-active proteins derived from a range of additional bacterial species. The proteins were classified according to the revised nomenclature rules (11).

## Database construction

The various functions of the website were implemented using the Django 2.2 framework and Python 3. The layout of the website was set up with the Bootstrap 4 package. JavaScript was used to complete the front-end functions, such as processing the data from the back-end, generating tables to display the data and generating the navigation bar to provide links to other pages. BLAST (13) and Needle (14) code was incorporated to allow protein comparison functionality. Code for Clustal Omega (15) was also included to allow the production of a guide tree; it was run with the following default parameters: gap penalty 10, gap extension penalty 0.20 and GONNET protein weight matrix. It should be noted that Clustal Omega uses the tree to guide the progressive alignment; it is not a reliable indication of the phylogeny of the sequences. The following JavaScript https://bl.ocks.org/git-ashish/3aa81521f96e48198c80b4e2742bb6bc was used to display the guide tree. The code for the database and associated tools can be found on GitHub https://github.com/bpprc.

## Pesticidal protein database

**Figure 2.**
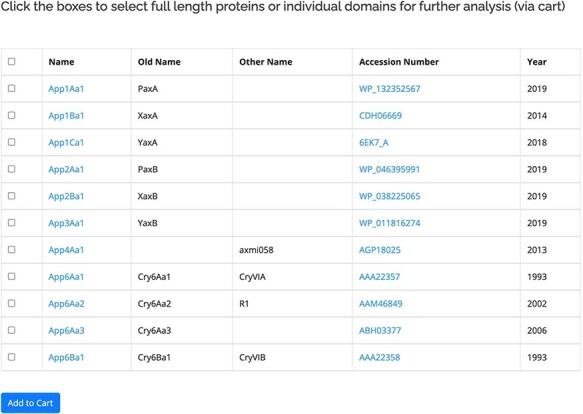
Primary user interface accessed via the *Search by name* option showing list of pesticidal proteins (App proteins in this example), along with old nomenclature and Accession numbers.

The proteins within the database are listed based on the revised nomenclature (11). The primary user interface for the proteins (Figure 2) displays the official name of the protein along with any previous or known alternative names. Also included in this view is a link to the GenBank entry for the protein and the year of the first report. A check box is provided to allow users to move selected sequences to the Cart for download or further analysis. For the 3-domain proteins, separate checkboxes are provided for the three individual domains of the active protein. The protein name is linked to more information about the protein, including its amino acid sequence. The sequences for some proteins in the database have not been released by GenBank, in which case the name will be available but related information cannot be accessed. The primary interface for the proteins can be accessed in two ways: by clicking on a structural class name, all the proteins within that class will be displayed. Alternatively, the search by name function can be used. This allows users to search for complete or partial names (e.g. Cry1Ac1 or Cry1A) as well as searching for specific accession numbers or alternative/old names. A separate database of pesticidal protein structures lists proteins for which structures have been experimentally determined (135 at the time of writing). This database is searchable by name and also by other fields including accession number and PDB_ID.

## Protein analysis

**Figure 3.**
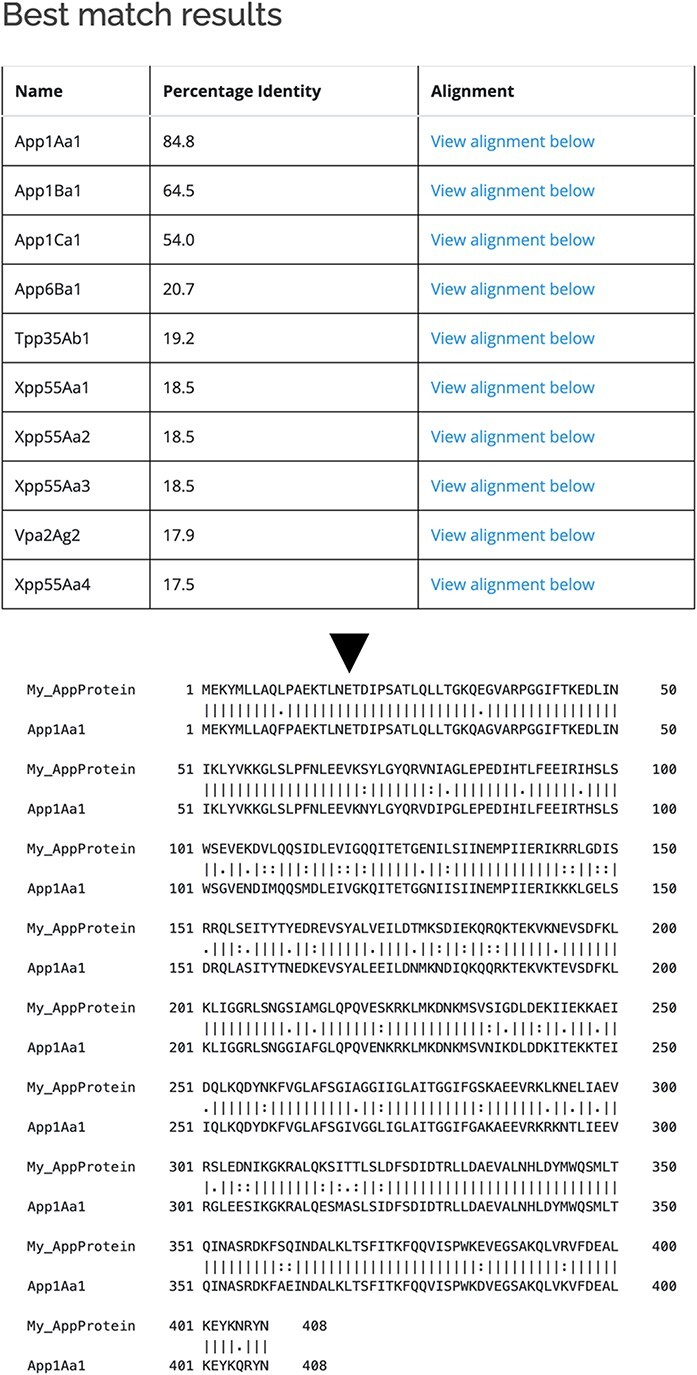
Use of BestMatchFinder tool with the *Search by sequence* option.

**Figure 4.**
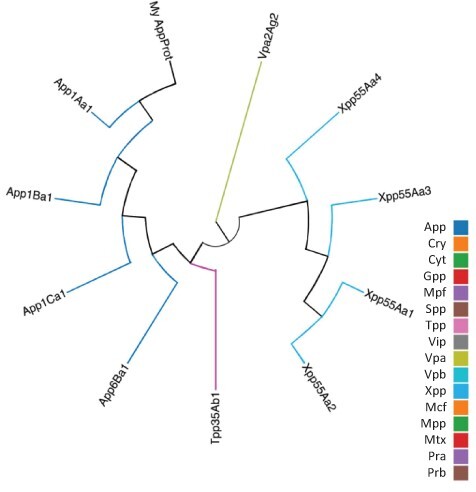
Guide tree showing relationships between a custom protein (My App protein) and proteins selected from the BPPRC database by the user.

As well as querying the database for particular proteins, users can search the database for those proteins with the highest sequence similarity to a manually entered sequence. This is achieved via a python script we have called BestMatchFinder (Figure 3). A sequence is entered in raw or fasta format and the algorithm returns a list of the top ten matches to sequences in the database along with a percentage identity value and a link to an alignment. Pairwise comparisons can also be performed between two proteins either from the database or user-defined. These comparisons can be undertaken using either the BLASTp (13) or Needle (14) algorithms. A third feature that we have incorporated is to produce a guide tree to show basic relationships between selected proteins. This is achieved by adding database or user-supplied sequences to the Cart and submitting the sequences for analysis (Figure 4). The resulting tree can be exported as a .pdf or a .png file.

## Submitting a sequence for naming

An online form is provided for users to submit sequences for naming. Within an admin section of the website, a python script applies the naming rules outlined in the nomenclature paper (11). Assuming that the protein satisfies the required criteria and following a manual check, the named sequence is uploaded to the database by a member of the nomenclature committee.

## Discussion and future work

The goal of the BPPRC is to provide a user-friendly interface to the revised pesticidal protein database and facilitate the incorporation of the diverse range of proteins being discovered largely as a result of genome sequencing projects. Scripts have been written to allow users to compare their own sequences to those in the database. Subsidiary databases can readily be added to the BPPRC database for pesticidal protein-related information. Metadata for the proteins, most notably data on pesticidal activities, are currently being added. This searchable functionality replaces the now defunct *Bacillus thuringiensis* toxin specificity database (https://cfs.nrcan.gc.ca/projects/119/2) and complements more specific projects such as ToxiTaxi (16) which looks at interactions between pesticidal proteins. Data from the BPPRC have also been integrated into other online resources such as BtToxin_Digger which screens genomic sequences for putative new pesticidal proteins (17). Future efforts may include a subdirectory on pesticidal protein receptors.

In the life sciences, the effective management of research data benefits the research community, industry, education and society. A report of the European Bioinformatics Institute showed that the benefit of effective research data management to researchers and funders exceeded 20 times the operational cost (18). As we continue to update and upgrade the resources available through BPPRC, the site will serve as an increasingly helpful tool to researchers and regulatory bodies around the globe in the field of pest control.
